# Biosynthesis of melatonin from l-tryptophan by an engineered microbial cell factory

**DOI:** 10.1186/s13068-024-02476-7

**Published:** 2024-02-18

**Authors:** Lijuan Wang, Yongdong Deng, Jianjie Gao, Bo Wang, Hongjuan Han, Zhenjun Li, Wenhui Zhang, Yu Wang, Xiaoyan Fu, Rihe Peng, Quanhong Yao, Yongsheng Tian, Jing Xu

**Affiliations:** 1 Shanghai Key Laboratory of Agricultural Genetics and Breeding, Biotechnology Research Institute of Shanghai Academy of Agricultural Sciences, 2901 Beidi Road, Shanghai, China; 2 Key Laboratory for Safety Assessment (Environment) of Agricultural Genetically Modified Organisms Ministry of Agriculture and Rural Affairs, 2901 Beidi Road, Shanghai, China

**Keywords:** Biosynthesis, Melatonin, l-Tryptophan (l-Trp), *Escherichia coli*, Metabolic engineering

## Abstract

**Background:**

The demand for melatonin is increasing due to its health-promoting bioactivities such as antioxidant and sleep benefits. Although melatonin is present in various organisms, its low content and high extraction cost make it unsustainable. Biosynthesis is a promising alternative method for melatonin production. However, the ectopic production of melatonin in microorganisms is very difficult due to the low or insoluble expression of melatonin synthesis genes. Hence, we aim to explore the biosynthesis of melatonin using *Escherichia coli* as a cell factory and ways to simultaneously coordinated express genes from different melatonin synthesis pathways.

**Results:**

In this study, the *mXcP4H* gene from *Xanthomonas campestris*, as well as the *HsAADC*, *HsAANAT* and *HIOMT* genes from human melatonin synthesis pathway were optimized and introduced into *E. coli* via a multi-monocistronic vector. The obtained strain BL7992 successfully synthesized 1.13 mg/L melatonin by utilizing L-tryptophan (l-Trp) as a substrate in a shake flask. It was determined that the rate-limiting enzyme for melatonin synthesis is the arylalkylamine N-acetyltransferase, which is encoded by the *HsAANAT* gene. Targeted metabolomics analysis of l-Trp revealed that the majority of l-Trp flowed to the indole pathway in BL7992, and knockout of the *tnaA* gene may be beneficial for increasing melatonin production.

**Conclusions:**

A metabolic engineering approach was adopted and melatonin was successfully synthesized from low-cost l-Trp in *E. coli*. This study provides a rapid and economical strategy for the synthesis of melatonin.

**Supplementary Information:**

The online version contains supplementary material available at 10.1186/s13068-024-02476-7.

## Introduction

Melatonin (N-acetyl-5-methoxytryptamine) is an important indoleamine that was initially isolated from the pineal gland in 1958 [1]. It is an endogenous biological clock regulator in the human body, and is mainly used clinically for the treatment of insomnia [2]. Not only this, but melatonin has potent antioxidant properties as a highly effective free radical scavenger, in addition to other biological activities such as anti-inflammatory, regulation of energy metabolism, skin protection and hypoglycemia [3–5]. Owing to the health benefits associated with melatonin, the demand for melatonin as a dietary supplement and medicine is increasing and the global market is expected to escalate from USD 1 billion in 2020 to USD 3.4 billion by 2026 [6]. Although melatonin is present in various organisms, including bacteria, insects, fungi, animals, and plants, its low levels make extraction low in purity, costly, and unsustainable [7]. Currently, melatonin is primarily produced through chemical synthesis, which has significant drawbacks such as cumbersome reaction steps and environmental pollution. For example, a classical method of preparing melatonin from 5-methoxyindole-3-acetonitrile requires four steps, and the process involves the use of toxic reagents such as pyridine, chloroform, and acetic anhydride [8]. Nonetheless, biosynthesis of melatonin, which requires only one step of fermentation and is environmentally friendly with no toxic solvents, may be the most promising alternative method for melatonin production [9].

With the development of molecular biotechnology, the biosynthetic pathway of melatonin in different species has been extensively studied. The classic pathway in mammals (including *Homo sapiens*) is deduced as follows: l-tryptophan (l-Trp) is initially transformed into 5-hydroxytryptophan (5-HTP) by the action of tryptophan 5-hydroxylase, after which it is converted into 5-hydroxytryptamine (5-HT, serotonin) through aromatic amino acid decarboxylase (AADC). 5-HT is then catalyzed by arylalkylamine N-acetyltransferase/serotonin N-acetyltransferase (AANAT/SNAT) and hydroxyindole-O-methyltransferase/N-acetylserotonin O-methyltransferase (HIOMT/ASMT) to generate the final product melatonin [10]. In plants, the first two steps of melatonin synthesis are reversed. l-Trp is first carboxylated to tryptamine by tryptophan decarboxylase and then catalyzed to 5-HT by tryptamine-5-hydroxylase [11]. Subsequently, in addition to SNAT and ASMT, the enzymes involved in the synthesis of melatonin from 5-HT also include caffeic acid O-methyltransferase (COMT), to replace the catalytic function of ASMT [12]. However, the melatonin synthesis pathway in microorganisms is poorly studied, and the genetic information involved in melatonin synthesis in microorganisms remains almost unknown.

Therefore, almost all the known genes related to the melatonin synthesis pathway are cloned from animals and plants [13, 14], their expressions in microorganisms are low or insoluble, particularly the ASMTs. Many animal ASMTs are insoluble and inactive expressed in *E. coli* [15]. Although some plant ASMTs can be expressed functionally, they show only low activities (0.29 and 0.21 pkat/mg protein) and even require GST tags to assist in soluble expression [16, 17]. So there are few reports about the ectopic production of melatonin in microorganisms [6, 13]. Byeon et al. [13] chose to clone a rice COMT gene to replace the function of ASMT, and reported the production of 1.46 mg/L melatonin in recombinant *E. coli* harboring sheep SNAT and rice COMT genes using relatively expensive serotonin as a substrate. Additionally, rice COMT displayed vastly different activities in the two dual-gene expression systems, indicating the complexity of multi-gene expression in *E. coli*. The COMT activity was subsequently improved by various means such as plasmid modification, alteration of gene insertion order in the vector, and site-specific mutation [18]. It can be seen that even in *E. coli*, a model strain with a clear genetic background and convenient tools for molecular cloning, the heterologous expression of melatonin synthesis pathway still faces many challenges.

In this study, we aimed to explore ways to biosynthesis of melatonin from l-Trp in engineered *E. coli*. To this end, a melatonin synthesis pathway containing four genes from *Xanthomonas campestris* and *H. sapiens* was designed and introduced into *E. coli* via a multi-monocistronic vector, and melatonin was successfully synthesized. The metabolism of l-Trp in *E. coli* was also analyzed to provide molecular modification targets for subsequent improvement of l-Trp utilization.

## Results and discussion

### Construction of a melatonin-producing engineered strain

**Fig. 1:**
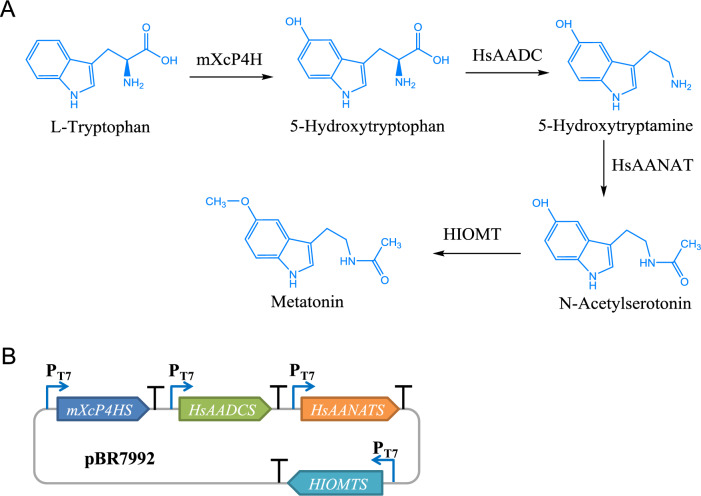
Biosynthesis of melatonin in *E. coli*. **A** Engineered pathway for the melatonin synthesis in BL7992. **B** The schematic representation of multi-monocistronic expression vector. *mXcP4H* tryptophan 5-hydroxylase, *HsAADC* aromatic amino acid decarboxylase, *HsAANAT* arylalkylamine N-acetyltransferase, *HIOMT* hydroxyindole-O-methyltransferase

*Escherichia coli*, as an appropriate substrate microorganism for industrial production, is an ideal host for melatonin biosynthesis. However, there have been only a few successful attempts at producing melatonin so far. To successfully synthesize melatonin in *E. coli*, coordinated expression of multiple genes of the melatonin biosynthesis pathway is required. First, a mutant P4H gene [19] with high activity in *E. coli*, and three other genes of human origin were selected based on the melatonin synthesis pathway in the human pineal gland (Fig. 1A). Second, the codons and mRNA structures of all four genes were optimized to improve transcriptional stability and translational efficiency. Third, a multi-monocistronic vector construction technique was used, in which each inserted gene was linked to the T7 promoter and terminator (Fig. 1B), to eliminate the weakening of expression levels caused by the long distance between promoters and gene sequences, and then to realize the coordinated expression of all the genes [20, 21]. In recent years, there have been some successful applications of multi-monocistronic vectors [20, 22]. Although protein levels are affected by the strength of transcription promoters, it has been shown that promoter strength affects RNA synthesis more than protein synthesis [23]. In order to establish a simple and general approach, the present study did not optimize promoters, but instead selected T7 promoter and terminator suitable for multi-gene expression to construct the multi-monocistronic vector pBR7992.The recombinant plasmid was subsequently transferred to *E. coli* BL21-AI (DE3) to obtain the engineered strain BL7992. The successful construction of the engineered strain was confirmed by PCR (Fig. 3A) and DNA sequencing.

**Fig. 2:**
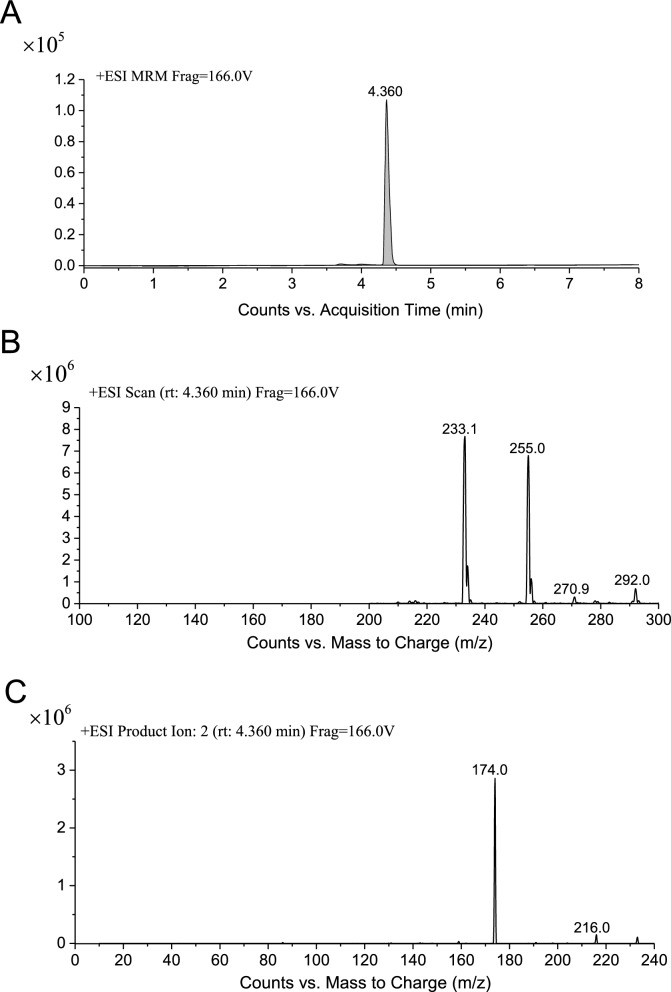
UPLC–MS/MS analysis of melatonin from BL7992. **A** Extracted ion chromatogram (XIC) of melatonin. **B** Primary mass spectrum of melatonin product (*m*/*z* (ES +): 232.1 [M + H]^+^). **C** Secondary mass spectrum of melatonin product (*m*/*z* (ES +): 174.0 [M + H].^+^)

The engineered strain BL7992 was cultured in shake flasks and the synthesis of melatonin was attempted by adding inducers (2 *g*/L arabinose and 1 mM isopropyl-β-D-thiogalactoside (IPTG)) and 1 *g*/L l-Trp as the substrate. The resulting supernatant was analyzed using UPLC–MS / MS to determine melatonin production. As shown in Fig. 2 and Additional file 1: Figure S1, a specific peak with the same retention time and mass spectrum as the melatonin standard was detected in the supernatant of BL7992 (*m*/*z* (ES +): 232.1 [M + H]^+^), and quantitative analysis indicated that this strain was capable of synthesizing 1.13 mg/L melatonin.

Here, we proposed a general workflow for the coordinated expression of multiple genes in *E. coli*, in which gene codons optimization and multi-monocistronic vector play an important role.

## Expression of exogenous genes in BL7992

**Fig. 3:**
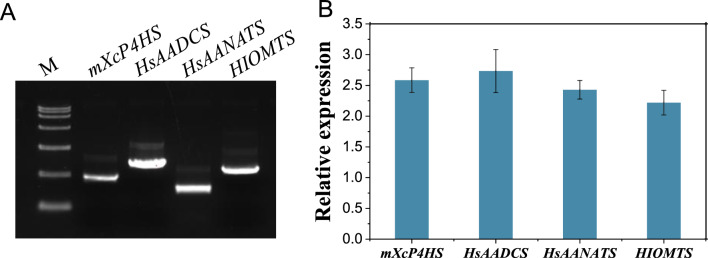
Cloning and expression analysis of the four synthetic genes in B7992. **A** PCR-amplified fragments using the plasmid extracted from BL7992 as template. M, DL15,000 marker. **B** The qPCR analysis using cDNA of BL7992 as template. The gene expression is relative to 16S rRNA expression

As the number of inserted genes increases, multi-gene vectors will become more complex and unstable [24]. In order to verify whether the functional enzymes were sufficiently and stably expressed in the transformants, the quantitative real-time polymerase chain reaction (qPCR) analysis was performed on all four genes. The results demonstrated that all of the genes were expressed at the transcriptional level, with only minor fluctuations in RNA quantity. As shown in Fig. 3, the *HsAADCS* gene exhibited the highest transcription level, 23% higher than that of the *HIOMTS* gene with the lowest transcription. Studies demonstrate that longer RNA molecules are more likely to fold and interfere with translation [25], and the insertion of multiple promoters and terminators can effectively avoid this phenomenon.

By coordinately expressing the four genes, BL7992 successfully synthesized melatonin (Fig. 2), indicated that the proteins encoded by these genes were capable of carrying out their respective functions, highlighting the effectiveness of the multi-monocistronic vector in multi-gene expression.

## Synthesis of melatonin in BL7992

**Fig. 4:**
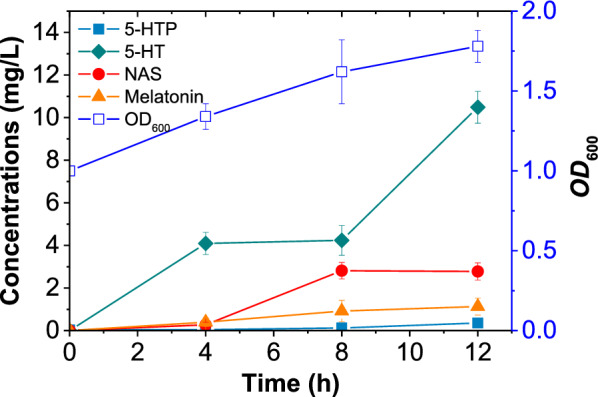
Time profiles of biomass (*OD*_600_) and the melatonin and intermediates concentrations in BL7992. 5-HTP, 5-hydroxytryptophan; 5-HT, 5-hydroxytryptamine; NAS, N-acetyl-5-hydroxytryptamine

The changes of intermediates during melatonin synthesis were tracked to elucidate the activities of individual enzymes and the rate-limiting step in the entire synthesis pathway. As shown in Fig. 4, the concentration of 5-HTP remained consistently low at all sampling points during the 12-h incubation period with the addition of l-Trp and inducers. While the content of 5-HT was the highest among all intermediates, accumulating to 10.5 mg/L at 12 h. It indicated that the conversion of 5-HT to NAS became the rate-limiting step for melatonin synthesis after 5-HTP was rapidly converted to 5-HT. Eventually, 1.13 mg/L of melatonin was synthesized in BL7992. Although this melatonin level was slightly lower than that reported by Byeon et al. (1.46 mg/L) [13]. The strain was able to produce melatonin using l-Trp as a substrate, which is much cheaper than 5-HT.

The expression activity of the enzymes was not consistent with the level of RNA transcription. Wang et al. [26] propose that protein production may be related to translation efficiency rather than mRNA concentration. In addition to protein content, protein activity is also affected by culture conditions such as induction temperature, which influences protein solubility [27]. It also can be seen that the activity of HsAANAT in *E. coli* was relatively low among the four enzymes involved in melatonin synthesis. This is one of the crucial factors responsible for the low melatonin synthesis concentration in BL7992. The AANAT reaction is also the main bottleneck in human melatonin biosynthesis [28]. It is therefore not surprising that the heterologous expression activity of HsAANAT in *E. coli* is weak. Enzyme activity of HsAANAT can be further improved by means of protein engineering and culture optimization [13, 29].

## Targeted metabolomics of l-Trp

It can be seen that with the addition of 1 *g*/L l-Trp as a substrate, the amount of each intermediate in the whole synthesis pathway is relatively small. In other words, only a very small proportion of l-Trp flowed into the exogenous melatonin synthesis pathway. l-Trp is not only an essential amino acid for protein synthesis in *E. coli*, but also a biosynthetic precursor for several metabolites [30]. Therefore, it is important to clarify the metabolic flux of l-Trp in recombinant strain to improve its utilization as a substrate for melatonin synthesis.

**Fig. 5:**
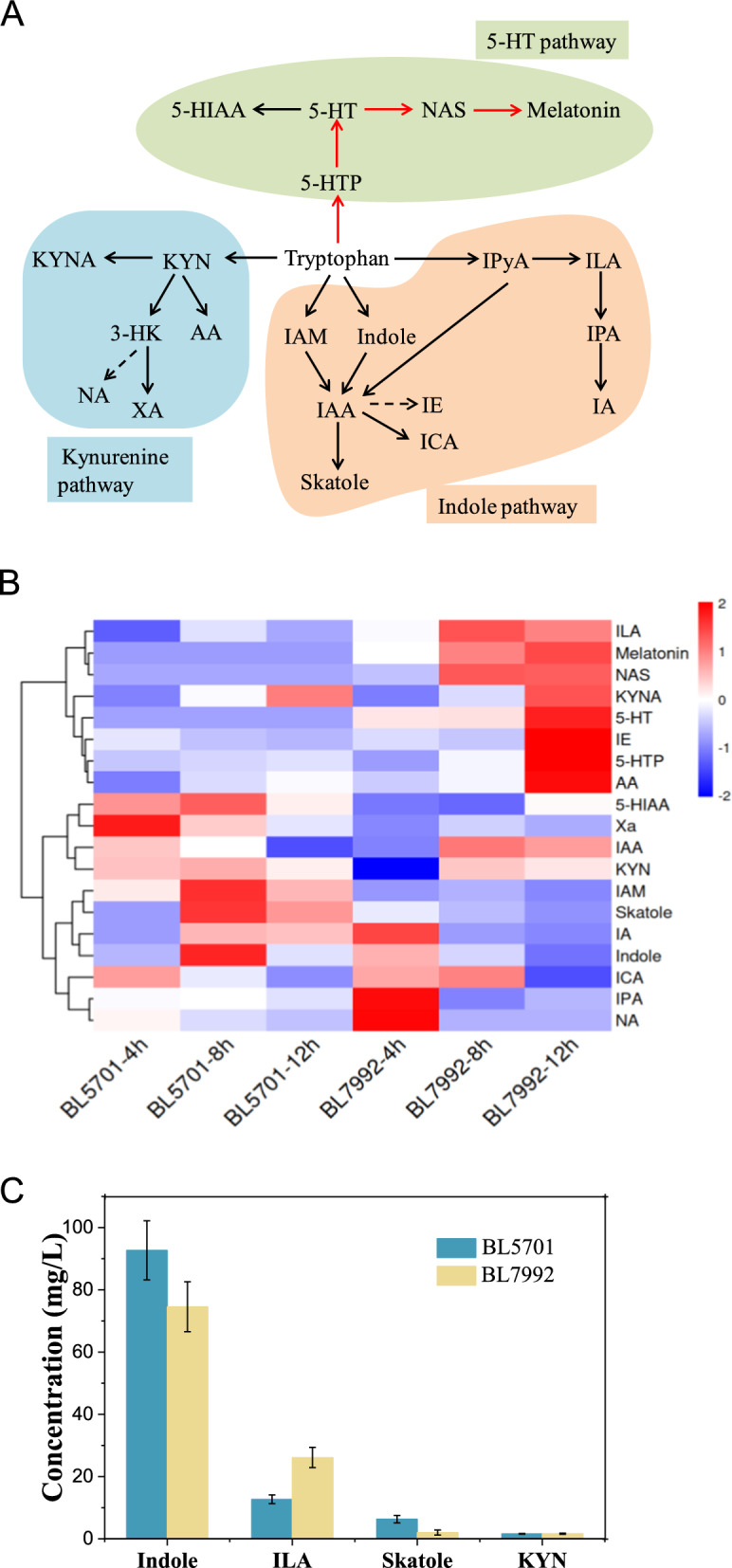
Effect of heterologous pathway on l-Trp metabolism. *5-HTP* 5-hydroxytryptophan, *5-HT* 5-hydroxytryptamine, *NAS* N-acetyl-5-hydroxytryptamine, *5-HIAA* 5-hydroxyindoleacetic acid, *IAM* indole-3-acetamide, *IAA* indole-3-acetic acid, *IE* tryptophol, *ICA* indole-3-carboxylic acid, *IPyA* indole pyruvic acid, *ILA* indole-3-lactic acid, *IPA* indole-3-propionic acid, *IA* indole acrylic acid, *KYN* kynurenine, *KYNA* kynurenic acid, *3-HK* 3-hydroxykynurenine, *AA* anthranilic acid, *XA* xanthurenic acid, *NA* nicotinic acid. **A** l-Trp degradation pathway in *E. coli*. ( →) native pathway, ( →) heterologous pathway. **B** Heatmap comparing the relative content of L-Trp metabolites between BL5701 and BL7992 with the addition of 1 *g*/L l-Trp (the darker the blue color, the lower the metabolite content, and the darker the red color, the higher the metabolite content). **C** The content comparison of the key metabolites between BL5701 and BL7992

Due to the introduction of exogenous melatonin synthesis pathway, the l-Trp metabolism pathway of BL7992 was more complex than that of control strain BL5701. In this study, UPLC–MS/MS was used to detect intermediate metabolites in the three major l-Trp metabolic pathways (5-HT pathway, kynurenine pathway and indole pathway) of the two strains (Fig. 5A). The targeted metabolomics were compared and analyzed. As shown in Fig. 5B, 12 h after the addition of l-Trp, the upregulated metabolites in BL7992 included indole-3-lactic acid (ILA), kynurenic acid (KYNA), tryptophol (IE), and anthranilic acid (AA) in addition to metabolites related to the 5-HT pathway. Down-regulated metabolites included indole, skatole, kynurenine (KYN), indole acrylic acid (IA), etc. The top 4 l-Trp metabolites are listed in Fig. 5C, and it shows that indole was the most abundant of all l-Trp metabolites tested, reaching 92.7 mg/L and 74.6 mg/L in BL5701 and BL7992, respectively. Although part of the l-Trp in BL7992 flowed to the melatonin synthesis pathway, resulting in a down-regulation of the indole, the level of indole (74.6 mg/L) was still fivefold the total concentration of all products in the melatonin synthesis pathway. Tryptophanase encoded by the *tnaA* gene catalyzes the degradation of l-Trp to indole, ammonium, and pyruvate [31]. The enzyme can be induced by high concentrations of exogenous l-Trp [32], leading to a significant accumulation of indole in the culture after the addition of 1 *g*/L l-Trp as a substrate. Additionally, the second most abundant intermediate, ILA, was also generated from the indole pathway. It can be concluded that the melatonin pathway was in a weak position in competition with the indole pathway. Although the KYN pathway is the main pathway for l-Trp metabolism in mammals, genes related to the KYN pathway are also contained in microorganisms [33, 34]. As shown in Fig. 5C, l-Trp flow to the KYN pathway in BL5701 and BL7992 was minimal and had little effect on l-Trp metabolism.

Briefly, a large amount of l-Trp flowed into the indole pathway, resulting in lower substrate utilization. The degradation of l-Trp to indole could be reduced by knocking out the *tnaA* gene in the indole pathway [35], thus making l-Trp more available for melatonin synthesis.

## Conclusions

In this study, the production of melatonin from l-Trp was achieved by expressing the melatonin synthesis pathway consisting of four genes from different sources. The engineered strain BL7992 was able to produce 1.13 mg/L melatonin without culture optimization. Although the melatonin yield of this strain is not yet competitive with chemically synthesized methods, it requires a less expensive substrate, l-Trp. The strain can produce melatonin through a simple one-step fermentation without the use of toxic solvents. The strain will be further modified by metabolic engineering, such as knocking out the *tnaA* gene, to improve l-Trp utilization and melatonin production. The present study provides a strategy for simple biosynthesis of melatonin from low-cost l-Trp.

## Materials and methods

### Chemicals, plasmids and strains

Molecular biology reagents were purchased from TaKaRa Biotechnology (Dalian) Co., Ltd. Other chemicals used in the study were purchased from Aladdin (Shanghai, China). The study complied with ethical and relevant guidelines for the use of molecular biological reagents and experimental procedures involving living organisms.

To improve the gene expression activity, *mXcP4H* (GenBank No. WP_011035413.1) (W179F mutant) from *X. campestris*, as well as the *HsAADC* (GenBank No. AY892283.1**)**, *HsAANAT* (GenBank No. BC069434.1) and *HIOMT* (GenBank No. U11091.1) genes from *H. sapiens* were optimized according to *E. coli* codon preference. The optimized genes are named as *mXcP4HS*, *HsAADCS*, *HsAANATS* and *HIOMTS*, and their sequences are listed in Additional file 1: Table S1. To construct a multi-monocistronic expression vector, the T7 promoter (5ʹ- CGATCCCGCGAAATTAATACGACTCACTATAGGGGAATTGTGAGCGGATAACAATTCCCCTCTAGAAATAATTTTGTTTAACTTTAAGAAGGAGATATA) and terminator (5ʹ-TAGCATAACCTTGGGGCCTCTAAACGGGTCTTGAGGGGTTTTTT G) were designed to the 5ʹ and 3ʹ ends of each gene, respectively. These expression cassettes were sequentially ligated to form a multi-gene expression cassette T7*mXcP4HS*–T7*HsAADCS*–T7*HsAANATS*–T7*HIOMTS*, and the *EcoR*I and *Hin*dIII digestion sites were designed at both ends of the sequence. The whole sequence was chemically synthesized and identified by Sangon Biotech Co. (Shanghai, China). The synthetic fragment was digested with *EcoR*I and *Hin*dIII before being inserted into pBR326 to obtain recombinant plasmid pBR7992. Subsequently, strain BL7992 was constructed by transferring pBR7992 to *E. coli* BL21-AI (DE3). Strain BL5701 (BL21-AI (DE3) harboring an empty vector pBR326 was used as control. Vector pBR326 was preserved in our laboratory [36]. BL21-AI (DE3) was purchased from Novagen.

### Shake-flask culture of the strains

The single colonies were incubated overnight at 37 ℃ in LB medium (10 *g* tryptone, 5 *g* yeast extract and 10 *g* NaCl per liter) supplemented with 50 mg/L kanamycin. Subsequently, a fresh seed culture of 1 mL was inoculated into a shaker containing 100 mL LB medium and cultured at 37 ℃ until the optical density at 600 nm (*OD*_600_) reached 1.0. The cells were harvested by centrifugation at 8000 rpm and suspended in 100 mL M9 minimal medium (3 *g* KH_2_PO_4_, 6 *g* Na_2_HPO_4_, 1 *g* NH_4_Cl, 0.5 *g* NaCl, 5 *g* acid-hydrolyzed casein, 0.5 mmol MgSO_4_, 0.1 mmol CaCl_2_ and 10 *g* glycerol per liter) containing 50 μg/mL kanamycin. At the same time, conventional doses of inducers (1 mM IPTG and 2 *g*/L arabinose) were added, as well as 1 *g*/L l-Trp was chosen to be added as a substrate after preliminary optimization, and cultivation was continued for a further 12 h at 37 ℃. All experiments were performed in triplicate.

### Gene expression analysis

The four genes involved in melatonin synthesis were amplified by PCR using the extracted plasmid as the template. The qPCR was utilized to investigate transcription levels of the exogenous genes. Total RNAs were extracted after 8 h of induction by TRIzol kit (Invitrogen) according to the manufacturer’s protocol, after which the cDNA was obtained by Takara RNA reverse transcription kit. The qPCR analysis was performed by Cobas z 480 analyzer (Roche Diagnostics) using cDNA as templates according to Wang et al. [37]. The specific primers used for PCR and qPCR are listed in Additional file 1: Tables S2 and S3, respectively. The relative expression values of genes were calculated by2^–ΔCT^ = 2^−[CT(target)−CT(16S)]^.

### Analytical methods

The cell biomass was determined by *OD*_600_ with a microplate reader (Tecan Infinite M200). For melatonin and the l-Trp catabolites analysis, 10 mL of fermentation broth was collected after 4 h, 8 h and 12 h of addition of substrate and inducer. The samples were then vacuum freeze-dried into lyophilized powder. Next, 10 mg of powder was mixed with 500 μL extract solution (methanol: acetonitrile: H_2_O = 2:2:1, containing 0.1% formic acid and internal standard). The samples were vortexed for 30 s, followed by homogenization for 4 min at 35 Hz and sonicated for 5 min in an ice-water bath. The extract was centrifuged at 12,000 rpm and 400 μL supernatant was taken, dried with nitrogen, and dissolved in 100 μL water containing 0.1% formic acid for detection. l-Trp catabolites were measured and quantified in mg/L by UPLC–MS/MS using an Exionlc System (Sciex), equipped with a Waters Acquity UPLC HSS T3 column (150 × 2.1 mm, 1.8 μm, Waters). The mobile phase was composed of solvent A (0.1% formic acid in water) and solvent B (0.1% formic acid in methanol). The gradient elution conditions were as follows: 0 – 6.5 min, 10 – 30% B; 6.5 – 7 min, 30 – 100% B; 7 – 14 min, 100% B; 14 – 17.5 min, 100 – 10% B. SCIEX Analyst Work Station Software (Version 1.6.3) and Sciex MultiQuant software (Version 3.0.3) were employed for MRM data acquisition and processing. The heatmap of l-Trp catabolites was performed using the bioDeep^™^ data analysis platform (http://www.biodeep.cn/).

### Supplementary Information

**Additional file 1: Table S1.** The gene sequences involved in this study. **Table S2.** Primers used for PCR. **Table S3.** Primers used for qPCR. **Figure S1.** UPLC–MS/MS chromatogram of the standard melatonin. Primary mass spectrum (*m*/*z* (ES+): 232.1 [M+H]^+^, secondary mass spectrum *m*/*z* (ES+): 174.0 [M+H]^+^.

## Data Availability

All data generated or analyzed during this study are included in this published article and its Additional information files.

## Abbreviations

5-HTP: 5-Hydroxytryptophan
5-HT: 5-Hydroxytryptamine
AADC: Aromatic amino acid decarboxylase
AANAT: Arylalkylamine N-acetyltransferase
SNAT: Serotonin N-acetyltransferase
HIOMT: Hydroxyindole-O-methyltransferase
ASMT: N-Acetylserotonin O-methyltransferase
COMT: Caffeic acid O-methyltransferase
KYNA: Indole, kynurenic acid
IE: Tryptophol
AA: Anthranilic acid
ILA: Indole-3-lactic acid
KYN: Kynurenine
IA: Indole acrylic acid
