# Supplementary material for: Biosynthesis of melatonin from l-tryptophan by an engineered microbial cell factory
Source: Biotechnol Biofuels Bioprod. 2024 Feb 18;17:27. doi: 10.1186/s13068-024-02476-7 (PMC10874579; doi:10.1186/s13068-024-02476-7)
Supplement: Supplementary file 1 — Additional file 1: Table S1. The gene sequences involved in this study. Table S2. Primers used for PCR. Table S3. Primers used for qPCR. Figure S1. UPLC–MS/MS chromatogram of the standard melatonin. Primary mass spectrum (m/z (ES+): 232.1 [M+H]+, secondary mass spectrum m/z (ES+): 174.0 [M+H]+. [file 13068_2024_2476_MOESM1_ESM.docx]

Supplementary Information

For

**Biosynthesis of melatonin from L-tryptophan by an engineered microbial cell factory**

Lijuan Wang^1,2†^, Yongdong Deng ^1,2†^, Jianjie Gao^1^, Bo Wang^1^, Hongjuan Han^1^, Zhenjun Li^1^, Wenhui Zhang^1^, Yu Wang^1^, Xiaoyan Fu^1^, Rihe Peng^1,2^, Quanhong Yao^1,2^, Yongsheng Tian^1,2*^, Jing Xu^1,2*^

^†^ Lijuan Wang and Yongdong Deng have contributed equally to the article

*Correspondence: tys810508@126.com; [xujingcn@yeah.net](mailto:xujingcn@yeah.net)

**^1^** Shanghai Key Laboratory of Agricultural Genetics and Breeding, Biotechnology Research Institute of Shanghai Academy of Agricultural Sciences, 2901 Beidi Road, Shanghai, China

**^2^** Key Laboratory for Safety Assessment (Environment) of Agricultural Genetically Modified Organisms Ministry of Agriculture and Rural Affairs, 2901 Beidi Road, Shanghai, China

**Table S1.** The gene sequences involved in this study

***mXcP4HS*** ATGAATACTGCACCACAACGTGTTGAGAATCAACTCACTGACAAAGGTTACGTTCCTGTCTACACTACTGCTGTTGTTGAACAACCTTGGGAATCATACTCTGCTGACGATCATGCAACCTGGGGTACTCTGTACCGTCGTCAGCGTGAGTTGCTGGTTGGTCGTGCATGTGATGAGTTCCTTCAGGCACAGGATGCAATGGGTATGGGTCAGACTCACATTCCACGTTTCGATGCACTCAATGAGGTCTTGCAAGCTGCAACTGGTTGGACTCTGGTTGGTGTTCAGGGTCTGCTGCCTGAGCTGGACTTCTTCGATCATCTGGCAAACAAGCGTTTCCCTGTCACCTGGTGGATTCGTCGTCCTGATCAGATCGACTACATTGCTGAACCTGACCTGTTCCATGATCTGTTCGGTCATGTTCCTCTGTTGATGAACCCACTGTTCGCAAACTTCATGCAGGCATACGGTCGTGGTGGTGTCAAGGCACATGGTATCGGTCCTGATGCACTCCAGAACCTGACTCGTCTGTACTGGTACACTGTTGAGTTCGGTTTGATCGATACTCCACATGGTCTGCGTATCTACGGTGCTGGTATCGTCTCATCCAAGGGTGAGTCACTGCACTCACTGGAGTCTGCTGCACCAAACCGTGTTGGTTTCGATCTGCAACGTATCATGCGTACTCGTTATCGTATCGACTCATTCCAGAAGACCTACTTCGTCATCGATTCCTTCGCACAGCTGATGGAAGCAACTGCACCTGACTTCACTCCAATCTACGCTGATCTGGCACAACAGACTCAGGTTCCTGCTGGTGATGTTCTGCCAACTGATCGTGTCATTCAACGTGGTTCTGGTGAGGGTTGGTCACGTGATGGTGATGTCTAA

***HsAADCS***

ATGAATGCATCTGAGTTCCGTCGTCGTGGTAAGGAGATGGTTGACTACGTTGCCAACTACATGGAAGGTATCGAAGGTCGTCAGGTCTACCCTGACGTTGAACCTGGTTATCTGCGTCCACTGATTCCTGCTGCTGCACCACAGGAACCTGACACCTTCGAGGACATCATCAACGATGTTGAGAAGATCATCATGCCTGGTGTCACTCACTGGCACTCTCCATACTTCTTCGCATACTTCCCAACTGCATCTTCCTATCCTGCAATGCTGGCTGACATGCTGTGTGGTGCAATCGGTTGCATTGGTTTCTCTTGGGCTGCATCTCCTGCATGTACTGAGCTGGAGACTGTCATGATGGACTGGCTGGGTAAGATGCTGGAACTGCCAAAGGCATTCCTGAACGAGAAGGCTGGTGAAGGTGGTGGTGTCATCCAGGGTTCTGCATCTGAAGCAACTCTGGTTGCACTGCTGGCTGCACGCACCAAGGTCATTCATCGTCTCCAGGCTGCATCTCCTGAACTCACTCAGGCTGCAATCATGGAGAAGCTGGTTGCCTACTCATCTGATCAGGCACACTCCTCTGTCGAACGTGCTGGTTTGATTGGTGGTGTCAAGTTGAAGGCAATCCCATCTGATGGTAACTTCGCAATGCGTGCATCTGCACTCCAGGAAGCACTGGAACGTGACAAGGCTGCTGGTCTGATCCCATTCTTCATGGTTGCAACTCTGGGTACTACTACCTGCTGCTCCTTCGACAACCTCTTGGAAGTCGGTCCAATCTGCAACAAGGAAGACATCTGGCTGCATGTTGATGCTGCCTATGCTGGTTCTGCATTCATCTGTCCTGAGTTCCGTCATCTCCTGAATGGTGTCGAGTTCGCTGACTCCTTCAACTTCAACCCACACAAGTGGCTCCTGGTCAACTTCGACTGCTCTGCAATGTGGGTCAAGAAACGCACTGACTTGACTGGTGCCTTCCGTCTGGACCCAACCTACCTGAAGCACTCACATCAGGACTCTGGTCTGATCACTGACTATCGTCATTGGCAGATCCCACTGGGTCGTCGCTTTCGTTCTCTGAAGATGTGGTTCGTCTTCCGCATGTACGGTGTCAAGGGTCTCCAGGCATACATTCGCAAGCATGTTCAGCTGTCCCATGAGTTCGAGTCACTGGTTCGTCAGGACCCACGCTTCGAAATCTGTGTCGAAGTCATCCTGGGTCTGGTCTGCTTCCGTCTGAAGGGTTCCAACAAGGTCAACGAAGCACTTCTGCAACGCATCAACTCTGCCAAGAAGATCCACCTGGTTCCATGTCACCTTCGCGACAAGTTCGTCCTTCGCTTCGCAATCTGTTCTCGCACCGTCGAATCTGCACATGTCCAGCGTGCATGGGAACACATCAAGGAACTGGCTGCTGACGTCCTGCGTGCTGAACGTGAGTAA

***HsAANATS***

ATGTCAACCCAATCCACTCATCCACTGAAACCTGAGGCACCACGTCTGCCACCTGGTATCCCTGAGTCACCATCTTGTCAGCGTCGTCACACTCTGCCTGCATCTGAGTTCCGTTGTCTCACTCCTGAGGATGCTGTCTCTGCATTCGAGATCGAACGTGAAGCATTCATCTCTGTCCTGGGTGTCTGTCCACTGTACCTGGATGAGATTCGTCACTTCCTCACTCTCTGTCCTGAACTGTCACTGGGTTGGTTCGAGGAGGGTTGTCTTGTTGCCTTCATCATTGGTTCACTCTGGGACAAGGAACGTCTCATGCAGGAGTCACTGACTCTGCATCGTTCTGGTGGTCACATCGCACATCTGCATGTTCTGGCTGTTCATCGTGCCTTCCGTCAGCAGGGTCGTGGTCCTATCCTGCTGTGGCGTTACCTGCATCATCTGGGTTCTCAACCTGCTGTTCGTCGTGCTGCACTCATGTGTGAAGATGCACTGGTTCCATTCTACGAACGTTTCTCATTCCATGCTGTTGGACCATGTGCCATCACTGTTGGTTCACTCACCTTCATGGAACTCCACTGCTCACTGCGTGGTCATCCATTCCTGCGTCGTAACTCTGGTTGCTAA

***HIOMTS***

ATGGGATCTTCTGAGGATCAGGCTTACAGACTCCTCAACGACTATGCTAACGGTTTCATGGTCTCTCAGGTCCTCTTCGCTGCTTGTGAGCTTGGTGTCTTCGACCTCCTTGCTGAGGCTCCAGGTCCACTTGATGTTGCTGCTGTTGCTGCTGGTGTCAGAGCTTCTGCTCACGGAACCGAACTCCTCCTCGACATCTGTGTCTCTCTCAAGCTCCTCAAGGTTGAGACCAGAGGAGGAAAGGCTTTCTACAGAAACACCGAACTCTCCTCTGACTACCTCACCACCGTCTCTCCTACCTCCCAGTGCTCTATGCTCAAGTACATGGGTAGAACCTCTTACAGATGCTGGGGACACCTCGCTGATGCTGTCAGAGAAGGTAGAAACCAGTACCTTGAGACCTTCGGTGTCCCTGCTGAAGAACTCTTCACTGCTATCTACAGATCCGAGGGTGAGAGACTCCAGTTCATGCAAGCTCTCCAGGAGGTCTGGTCTGTCAATGGTAGATCCGTCCTCACTGCTTTCGACCTCTCCGTCTTCCCACTCATGTGTGACCTCGGTGGAACCAGAATCAAACTTGAGACCATCATCCTCTCTAAGCTCTCTCAAGGACAGAAGACCAAGCACAGAGTCTTCTCTCTCATCGGTGGTGCTGGTGCTCTCGCTAAGGAGTGCATGTCTCTCTACCCTGGATGCAAGATCACCGTCTTCGACATCCCTGAAGTTGTTTGGACTGCTAAGCAGCACTTCTCTTTCCAGGAGGAAGAACAGATCGACTTCCAGGAAGGTGACTTCTTCAAGGACCCACTGCCTGAAGCTGACCTCTACATCCTTGCTAGAGTTCTCCACGATTGGGCTGATGGAAAGTGCTCTCACCTCCTTGAGAGAATCTACCACACTTGCAAGCCAGGTGGTGGTATCCTCGTCATCGAGTCTCTCCTCGACGAAGACAGAAGAGGTCCACTCCTCACCCAGCTCTACTCTCTCAACATGCTCGTCCAGACCGAAGGACAGGAGAGAACTCCAACCCACTACCACATGCTCCTCTCTTCTGCTGGATTCAGAGACTTCCAGTTCAAGAAGACTGGAGCTATCTACGACGCTATCTTGGCTAGAAAGTAA

**Table S2.** Primers used for PCR

| **Name** | **Premer sequence (5’ to 3’)** |
| --- | --- |
| *mXcP4HS*-F1 | ATGAATACTGCACCACAACGTG |
| *mXcP4HS*-R1 | TTAGACATCACCATCACGTGAC |
| *HsAADCS*-F1 | ATGAATGCATCTGAGTTCCGT |
| *HsAADCS*-R1 | TTACTCACGTTCAGCACGCAGG |
| *HsAANATS*-F1 | ATGTCAACCCAATCCACTCATC |
| *HsAANATS*-R1 | TTAGCAACCAGAGTTACGACGC |
| *HIOMTS*-F1 | ATGGGATCTTCTGAGGATCAGG |
| *HIOMTS*-R1 | TTACTTTCTAGCCAAGATAGCG |

‘

**Table S3.** Primers used for qPCR

| **Name** | **Premer sequence (5’ to 3’)** |
| --- | --- |
| *mXcP4HS*-F2 | TGTTGAACAACCTTGGGAATC |
| *mXcP4HS*-R2 | TCCACCAGGTGACAGGGAAAC |
| *HsAADCS*-F2 | AGATCATCATGCCTGGTGTCA |
| *HsAADCS*-R2 | CAGCCAGCAGTGCAACCAGAG |
| *HsAANATS*-F2 | CACTGTACCTGGATGAGATTC |
| *HsAANATS*-R2 | TGCAGCACGACGAACAGCAGG |
| *HIOMTS*-F2 | ACAGAAACACCGAACTCTCCT |
| *HIOMTS*-R2 | GAGGTCGAAAGCAGTGAGGAC |

**Figure S1.** LC-MS/MS chromatogram of the standard melatonin. Primary mass spectrum (m/z (ES+): 232.1 [M+H]^+^, secondary mass spectrum m/z (ES+): 174.0 [M+H]^+^.
